# Sand Fly Fever with Different Names

**Published:** 2017-03-14

**Authors:** Ali Mehrabi-Tavana

**Affiliations:** Health Management Research Center, Baqiyatallah University of Medical Sciences, Tehran, Iran

**Dear Editor-in-Chief**

**With regard to a paper published in Journal of Arthropod-Borne Diseases;**

A Case Report entitled “Sand fly Fever with Skin Lesions: A Case Series from Turkey” was published in 2016 (Fatih Temocin, et al. 2016). Different names such as “*Phlebotomus* fever”, ‘’mosquito fever’’, three-day fever or “Papatasi fever” called the disease of Sand fly fever which all are true except ‘’mosquito fever’’ (Tavana 2001, 2015). Mosquito is not a general name in English and is used just for Culicidae. Blackflies and biting midges are used for Simuliidae and Ceratopogonidae, respectively. Therefore, is not related to sand fly fever (Saidi et al. 1977, Izri et al. 2008). In my opinion it should be corrected. Sand fly fever is an arthropod-borne viral disease, starts with acute onset of high fever, and lasts for two to four days, sometimes much longer.

Photophobia and muscle ache are more common in the patients. This disease is more prevalent in Europe, Asia, and Persian Gulf region (Konstantinou et al. 2007, Tavana 2007). It is important to be aware and take care of this disease in endemic and no-endemic area among travelers returning from endemic areas.
